# Government (Teaching) and Municipal (Non-Teaching) German Hospitals


**Published:** 1912-11-02

**Authors:** Henry Burdett


### GOVERNMENT (Teaching) and MUNICIPAL (Non-Teaching) GERMAN HOSPITALS.*

By SIR HENRY BURDETT, K.C.B.

### IV. State versus Voluntary Hospitals.

An interesting and sound article under this heading, based upon the first of these reports, appeared in our contemporary the *Queen* of October 26. It properly rubs in the fact that in this country, where freedom has continued so unchecked for centuries that to-day it militates against liberty, and is apt to degenerate into licence, it is certain that if any Government attempted to supplant the voluntary hospitals by establishing a system of State hospitals, with the official and disciplinary routine which must dominate such institutions, the people of all classes would rise in rebellion, for they would never consent to become mere material for science. Further, they cannot afford to relinquish the tender, loving care and personal service in hospitals which is guaranteed to the English people of all classes under the voluntary system. It is well—nay, it has become necessary—however, to raise a note of caution and to stimulate to prompt action the responsible managers of every voluntary hospital throughout the United Kingdom.

### The Pending Hospital Crisis.

We Britons are within measurable distance of a great crisis with regard to medical relief, for we are confronted with the momentous fact that on and after January 15, 1913, 15,000,000 people or thereabouts will become entitled as insured persons to medical benefits under the National Insurance Act. It is estimated by probably the ablest and most knowledgeable of all the insurance managers that of this number at least 600,000, or four per cent., will at once require medical benefit. Further, that the proportion of those requiring such benefits week by week will tend to increase in numbers and not to diminish. Of this weekly minimum of 600,000 persons a large proportion will need and must somehow obtain hospital treatment. Mr. Lloyd George, although the matter was brought urgently to his notice and specially represented to the Cabinet, decided not to provide for the hospital requirements of insured persons, but to ignore them altogether. Further than this, no agreement or arrangement has been made at present under the Act for the provision of medical benefit, even apart from the hospital treatment which the Government have deliberately ignored. It is to be hoped, of course, that Mr. Lloyd George and the members of the medical profession may arrive at some working settlement before January 15 next. But, assuming that they do, definite hospital treatment for insured persons will still remain non-existent and unprovided. It is perfectly clear that the voluntary hospitals cannot without grave abuse grant free medical relief to insured persons and so put them in the humiliating position which their German confrères have refused to occupy, that of being recipients of charitable relief.

### Medical and Business Aspects.

But, apart from the question of the degradation of the insured, there remain the medical and business aspects to be considered. It has become perfectly clear that the decision arrived at by the meeting of the metropolitan hospitals managers, held in January 1912, to let matters slide for a year by ignoring altogether whether patients seeking admission to hospitals are insured persons or not, cannot be acted upon. Up and down the country reports have been published of communications received by the responsible managers of great voluntary hospitals from the members of their medical staff, intimating that after January 15 next they will not be prepared to give free medical service to any in- or out-patient at a voluntary hospital, being an insured person. In another column, p. 115, we give a striking example from Portsmouth of the refusal of the leading members of the medical profession to grant free medical relief to such insured persons who present themselves as patients for free treatment at a voluntary hospital. This is essentially an emergency when a conference of all the managers of voluntary hospitals throughout the country should be summoned, without delay, to a meeting whereat this pressing problem can be fully discussed and arrangements made to appoint the most representative, important, and largest deputation which has ever yet been organised, to seek an interview with the Chancellor of the Exchequer or the Prime Minister, or both. This deputation should demand that the interests of this enormous army of sufferers, some of whom have heretofore had hospital care, as and when they have needed it, shall not suffer because the Government of the day has legislated in haste, and refused to face the pressing hospital needs of the population. All the facts were brought forcibly to the Government's notice before the Act was passed, yet with the knowledge of the certainty of the crisis which has actually arisen, they deliberately refused to provide against it. In any case the refusal of Mr. Lloyd George to give adequate time to enable the many momentous questions and difficulties created by the introduction of a great system of national insurance into this country to be threshed out, of which the hospital question is but one, has resulted in making it imperative that, not only the managers of the voluntary hospitals, but every thinking man and woman throughout the United Kingdom shall promptly consider the relative merits of State and voluntary hospitals. If they do so with knowledge, they will speedily combine to ensure for themselves and their families the perpetuation in this free country of the safeguards and

* Previous articles of this series appeared in our issues of October 12, 19, and 26.

privileges which they and their children have enjoyed under the voluntary system of hospitals. This system at its best is undoubtedly the most humane, the least extravagant, and most efficient hospital system the world has ever seen.

### The Experience of Germany.

In order to understand and appreciate the present position of State and municipal hospitals in Germany it is essential to realise the enormous changes which have resulted from the creation of the Empire under the Constitution of April 16, 1871. At that period, we can say without offence, for it has been shown to be so by some of the most knowledgeable and able of the German authorities, that the hospitals which were then in existence in the various states, kingdoms, and cities of the countries which now constitute the German Empire, left much to desire. As we pointed out in an earlier article, no self-respecting member of the working or middle classes in Germany would then enter one of the general hospitals, which, as we have said, were regarded as pauper asylums or homes for the dying. The prejudice was also great against the charitable hospitals or religious houses, the management of which was not generally approved and admission to which was regarded as the acceptance of charity, an indignity which no member of the German artisan or middle classes would suffer. In matters of construction, routine, and general administration they did not approach the standard of the better type of hospitals to be met with in other countries. It naturally took years for the German people to pull themselves together and gradually to awaken to the immense possibilities which the new Constitution offered to all classes of the people. This period of awakening and settlement roughly extended from 1871 to 1884—fourteen years—not a long period either in the circumstances. Then, as Ober-Inspektor Einert, one of the most knowledgeable in the administrative sense of hospital officers in Germany to-day, has pointed out, a complete change in the attitude towards hospitals of the public, the medical profession, and the imperial, State, and municipal Governments began to show itself. In 1884 the hospitals of Germany and the position taken up by the artisan and middle classes towards them began to change. This altered spirit during the last twenty-five years has revolutionised hospital provision, hospital treatment, and the opinions and feelings of all classes of Germans in regard to both. One result has been that to-day the private general hospitals which still exist are due to special conditions or historical circumstances, and not to any great belief or desire of the people of any class to provide hospital accommodation on a charitable basis. There are, of course, important Imperial and State hospitals which have been radically changed, reconstructed, and improved in all respects during this period where the bulk of the medical instruction is centred. The majority of the hospitals throughout Germany to-day are mostly vested in the municipality, and not in State or private corporations. One striking evidence of the enormous change in public opinion and of the improved prosperity and development of the German people throughout the Empire is the fact that the great majority of municipalities have taken the maintenance of public hospitals on to their own shoulders. So general has been the adoption of this principle that the ratepayers now hold that hospital provision of the highest type is one of the compulsory duties of each municipality, and that it constitutes an important portion of the necessary measures municipalities have to take for the welfare and health of the people.

### The Awakening of the German People.

In the first article of this series we testified to the many excellences which the best municipal hospitals exhibit as a result of this awakening of public opinion, and the pride which the Germans are exhibiting in ever-increasing measure in the establishment and maintenance of every kind of educational and hygienic provision which tends to secure the intellectual development, the physical welfare, and the highest standard of efficiency in the best interests of the municipality, the State, and the Empire. How universal has been the awakening of the German people under the Empire, and how deep-rooted has become the spirit of patriotism and of satisfaction with the openings and opportunities for individual advancement and family comfort now provided throughout Germany, is strikingly illustrated by the changed conditions of emigration from Germany now and before the Empire was established in 1871. Out of 30,000,000 of emigrants from Europe in the nineteenth century Germany contributed about 5,000,000, or one-sixth of the whole number. Ten years after the foundation of the Empire—*i.e.* in 1881—upwards of 220,000 German emigrants left their native country. At the period of the great awakening to which we have referred—*i.e.* in 1886—there were only 83,000 German emigrants, and since that time the number has steadily decreased, until the Germans who emigrated in 1910 amounted to only 25,531 souls. Some politicians in this country have made a great point of the miserable conditions under which the artisans of Germany live, and of the black bread they eat. These figures eloquently testify that the comments in question belong in reality to a type of political lie which makes politics nowadays so distasteful to the majority of thoughtful persons of all classes.

### What English Men and Women will Never Suffer.

We must take it, however, in view of the facts here produced, that there never was a time when the people of Germany were prouder of or more contented with their country or the prospects which it offers to all classes for advancement and comfort than they are to-day. We can testify after full inquiry, by the production of evidence, facts, and figures in our possession, by a personal inspection of the most representative and principal municipal hospitals Germany contains, that the whole trend of advancement in Germany in regard to hospitals, so far as their hygienic, medical, and administrative equipments and technical efficiency are concerned, is high, and tends every day to increase rather than to diminish. On these points there is small room, if any, for differences of opinion. But when all this has been said, and the fullest recognition has been given to the relatively high technical efficiency of the best hospitals of Germany at present, we are bound to testify that English men and women would never rest content to be patients in the general wards of institutions, under an iron discipline, which deprives the patients in a hospital of the safeguards of an independent and articulate public opinion, and ministers to the setting back of humanity and the infinite lessening of the humanising influences without which the spirit of personal service and trained nurses cannot be maintained, nor the delightful atmosphere which is to be found to-day in the best-administered of our voluntary hospitals throughout Great Britain. It follows that the more knowledge the people of these islands gain of a patient's life in municipal hospitals conducted on the foreign principle, where scientific treatment is apt to control affairs completely, the more certain it is that a great system of State and municipal hospitals will never be set up in Great Britain to the exclusion or destruction of the voluntary hospitals, which confer such infinite blessings upon all classes at the present time. But, apart from questions of humanity and all questions of comfort, adequate nursing, and attraction for the patients, we shall show next week that, as a matter of business, the cost of these great municipal hospitals is becoming so onerous that German opinion is looking with longing eyes in the direction of the voluntary system of hospital support, with the hope and intention of relieving the municipalities of a portion at any rate of the burden which the awakening of German opinion has imposed upon the ratepayers by demanding in every great city a system of modern hospitals of the highest technical efficiency.

